# Acromioclavicular third degree dislocation: surgical treatment in acute cases

**DOI:** 10.1186/s13018-014-0150-z

**Published:** 2015-01-28

**Authors:** Angelo De Carli, Riccardo Maria Lanzetti, Alessandro Ciompi, Domenico Lupariello, Pierpaolo Rota, Andrea Ferretti

**Affiliations:** Orthopaedic Unit and “Kirk Kilgour” Sports Injury Centre, S. Andrea Hospital, University of Rome “La Sapienza”, Italy, Via di Grottarossa 1035, 00189 Rome, Italy

**Keywords:** Acromioclavicular joint dislocation, TightRope, Rockwood type III dislocation, Shoulder

## Abstract

**Background:**

The management of acute Rockwood type III acromioclavicular joint (ACJ) dislocation remains controversial, and the debate about whether patients should be conservatively or surgically treated continues. This study aims to compare conservative and surgical treatment of acute type III ACJ injuries in active sport participants (<35 years of age) by analysing clinical and radiological results after a minimum of 24 months follow-up.

**Methods:**

The records of 72 patients with acute type III ACJ dislocations who were treated from January 2006 to December 2011 were retrospectively evaluated. Patients were categorised into two groups. group A included 25 patients treated conservatively, and group B included 30 patients treated surgically with the TightRope™ system. Seventeen patients were lost to follow-up.

All patients were evaluated at final follow-up with these clinical scores: Constant, University of California Los Angeles scale (UCLA), American Shoulder and Elbow Surgeons Scale (ASES) and Acromioclavicular Joint Instability (ACJI) and with a subjective evaluation of the patient satisfaction, aesthetic results and shoulder function. The distance between the acromion and clavicle and between the coracoid process and clavicle were evaluated radiographically and compared with preoperative values. Δ, the difference in mm between the distance at the final follow-up and at T0 in the injured shoulder, and *α*, the side-to-side difference in mm at follow-up, were calculated. Heterotopic ossification and postoperative osteolysis were evaluated in both groups.

**Results:**

There were no major intraoperative complications in the surgical group. The subjective parameters significantly differed between the two groups. Constant, ASES and UCLA scores were similar in both groups (*P* > 0.05), whereas ACJI results favoured the surgical group (group A, 72.4; group B, 87.9; *P* < 0.05). All measurements of radiographic evaluation were significantly reduced in the surgical group compared with the conservative group. In group A, we detected calcifications in 30% of patients; in group B we detected two cases of moderate osteolysis and calcifications in 70% of patients.

**Conclusion:**

Although better subjective and radiographic results were achieved in surgically treated patients, traditional objective scores did not show significant differences between the two groups. Our results cannot support routine use of surgery to treat type III ACJ dislocations.

## Background

Acromioclavicular joint dislocation is one of the most common shoulder injuries treated in general orthopaedic practice. It is the most frequent shoulder injury among contact sport participants [1]. Acromioclavicular dislocations occur in 41% of collegiate football players and in 40% of (US) National Football League quarterbacks [2,3]. These dislocations are more common in men than in women, at a five to one ratio [4], perhaps because men are more likely to practise contact sports than women are.

There is agreement about conservative treatment for types I and II ACJ dislocations [5-7], whereas surgical treatment seems to be the best choice for types IV, V and VI ACJ dislocations [8-11]. Surgical management for acromioclavicular dislocation has been advocated because it restores joint anatomy, thus avoiding obvious deformity and a potentially unsatisfactory outcome [12]. However, disadvantages of surgery include migration of pins used for fixation, erosion of the bone by fixation devices, failure of metallic fixation devices, recurrence of deformity, a painful or unsightly scar, late development of acromioclavicular pain and arthritis and a mandatory second operation to remove fixation devices [13]. The advantages of conservative treatment include shorter rehabilitation and sparing hospitalisation [12]. The reported disadvantages of conservative treatment include unsatisfactory results in approximately 20% of patients due to pain, instability and limitation of motion [14].

The gold standard for the treatment of acute acromioclavicular joint (ACJ) Rockwood type III dislocation is still debated. Most authors obtained good-to-excellent results with nonsurgical management of patients with type III injuries [5,15,16]; however, others have reported persistent pain and residual symptoms associated with the acromioclavicular joint at final follow-up evaluations [17-19]. Thus, to improve functional results, some authors advocate surgery for acute type III acromioclavicular joint injuries in young and active patients [20-22]. Moreover, recently, Kirsten et al. reported that in young active patients with type III ACJ dislocation, surgical treatment seems to offer better subjective and cosmetic results than does conservative treatment [23]. The hypothesis of our study was that surgical treatment achieves better results than conservative treatment in athletes between 18 and 35 years old with type III dislocation. This study aimed to compare conservative and surgical treatment of acute type III ACJ injuries in these athletes by analysing clinical and radiological results after a minimum of 24 months follow-up.

## Methods

A retrospective study of patients with acute Rockwood type III acromioclavicular dislocation who were diagnosed and treated at our Orthopaedic Department between January 2006 and December 2011 was performed. Inclusion criteria were age between 18 and 35 years, injury occurred within 21 days [24], absence of concomitant injury or previous surgery and absence of concomitant acromial, coracoid or clavicular fracture. Seventy-two patients met our inclusion criteria, but 17 were lost to follow-up. The remaining 55 patients were categorised into two groups: group A, 25 patients treated conservatively with 4-week immobilisation using a Kenny-Howard brace and group B, 30 patients treated surgically with the TightRope™ system (Arthrex, Naples, USA). That system consists of a clavicular round (6.5 mm in diameter) and an oblong coracoid titanium button (10 × 3.5 mm) connected by nonabsorbable No. 5 Fiberwire suture (Arthrex) [25].

After the risks and benefits of conservative or surgical treatment were discussed with the surgeon, the decision between an operative or conservative procedure was made by the patient. Before undergoing treatment, written informed consent was provided by all patients. Both groups of patients underwent clinical and radiological examination at follow-up.

### Clinical evaluation

For the clinical and functional objective evaluation, we used four validated international scales: the University of California Los Angeles shoulder rating scale (UCLA) [26], which analyses pain, range of motion (ROM), function, strength and satisfaction; the American Shoulder and Elbow Surgeons Scale (ASES) [27], which analyses pain and daily life activities; The Constant Score [28], which includes pain, function, ROM and strength; and the Acromioclavicular Joint Instability Scoring System (ACJI) [25], which includes pain, daily life activities, cosmesis and radiological assessment.

In addition, a subjective evaluation was performed at final follow-up. The patient was asked to a subjectively evaluate the injured shoulder as excellent, good, fair and poor. Moreover, they were asked about their cosmetic subjective satisfaction expressed as highly satisfied, satisfied, poorly satisfied and unsatisfied. Finally, the patient was asked to assign a percentage to the injured shoulder function compared to the contralateral healthy shoulder (Subjective Shoulder Value, SSV).

### Radiological evaluation

**Figure 1:**
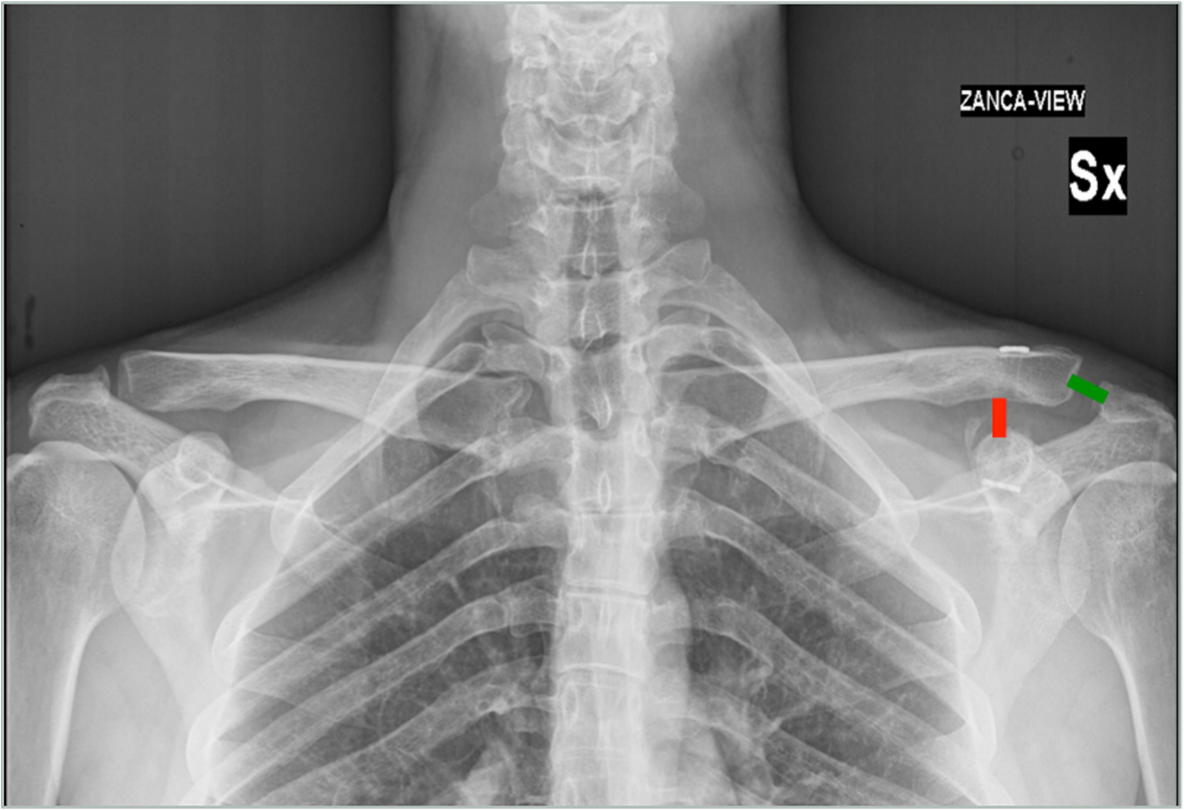
**Radiological examination.** Green line shows the distance between the acromion and lateral clavicle (ACD); red line shows the distance between the coracoid process and clavicle (CCD).

The radiological examination consisted of AP and axillary radiographs for each shoulder and bilateral stress radiographs [29]. At the final follow-up visit, an Alexander view radiograph was obtained to calculate the ACJI score as described by Scheibel et al. [25]. The distance between the acromion and lateral clavicle (ACD) and the distance between the coracoid process and clavicle (CCD) were measured (Figure 1). The ACD was measured between the centre of the medial aspect of the acromion and the centre of the lateral aspect of the clavicle. The CCD was measured between the coracoid and inferior cortex of the clavicle [30]. All measurements were performed on the injured and healthy side in stress radiographs at admission (T0) and at the final follow-up visit.

In addition, these parameters were calculated:

- Δ: the difference in mm between a distance (CCD or ACD) measured at the final follow-up and this distance measured at T0 in the injured shoulder.
- *α*: side-to-side difference in mm of a distance measure at follow-up.

Heterotopic ossification and postoperative osteolysis were also evaluated in both groups [31].

### Surgical treatment

The mean interval between injury and operation was 7 days (range 0–15 days). General anaesthesia was used in all cases and patients were placed in the “beach-chair” position. An anterior approach was performed in all cases, using a vertical skin incision toward the coracoid tip, starting at the posterior edge of the lateral clavicle (mean scar length, 3.9 mm). The fascia of the deltoid muscle was opened along the fibre and the superior part of the distal clavicle and the acromioclavicular joint line were exposed. The base of the coracoid was identified. After exposing the clavicle and coracoid process, a 4-mm hole was drilled into the clavicle approximately 40 mm from the lateral clavicular edge of the coracoids and into the coracoid base to allow placement of the TightRope. The reduction was performed under visual control. The mean operative time was 42 min (range, 37–49 min).

Postoperatively, the shoulder was protected for 4 weeks using an arm immobiliser sling. However, pendular exercises were commenced within 2 weeks after the first visit. At 4 weeks, passive and active assisted range-of-motion (ROM), isometric and closed chain exercises were begun. Active exercises through full ROM (ensuring scapula dynamic control) in open chain (below the coronal plane) were started 8 weeks postoperatively. Contact sports and heavy work were permitted after 12 weeks.

### Conservative treatment

The shoulder was placed in a Kenny Howard brace. During the first postoperative days, an ice pack was applied, and mild analgesics were given to the patient. Pendular exercises were permitted after 2 weeks. After 4 weeks, the brace was removed and shoulder motion was progressively increased as symptoms permitted, initially using closed chain exercises and later using active exercises in open chain with the aim of recovering strength and range of motion.

### Statistical analysis

Statistical analysis was performed with SPSS (version 11.5.1). Continuous variables were compared using the Student *t*-test. Results were analysed and the study groups were compared with each other. The chi-square test was used to detect the impact of each procedure. Continuous variable were described using the mean ± sd. The level of significance was set as *P* = 0.05. Illustrations were created using SPSS (version 11.5.1).

## Results

All patients were male with a mean age at the time of this study of 28.7. The mean follow-up time, 3.5 years (range, 2–8 yrs), did not differ significantly between the two groups. All patients were recreational athletes (15 rugby players, 14 soccer players, 5 handball players, 3 basketball players, 4 volleyball players, 9 bikers, and 5 cyclists; the frequency of these sports did not differ by group. Ten patients were sedentary workers, and 20 patients performed light work, 20 performed medium work and 5 heavy work; the frequencies of these work categories were similar between groups.

### Clinical evaluation

The clinical assessment using our validated international scales showed the following results. The mean UCLA scores, 33.5 ± 1.7 in group A and 34 ± 0.9 in group B, were not significantly different, *P* > 0.05; the mean Constant scores, 98% ± 3.2 in group A and 98.2% ± 2.8 in group B, were not significantly different, *P* > 0.05; and the mean ASES scores, 98.5 ± 1.6 in group A and 100 in group B, were not significantly different, *P* > 0.05. In contrast, the mean ACJI scores, 72.4 ± 1.8 in group A and 87.9 ± 2.2 in group B, were significantly different, *P* < 0.05 (Table 1).

**Table 1:** **Objective evaluation**

	**Group A**	**Group B**
Constant	98%	98.2%
UCLA	33.5	34
ASES	98.5	100
ACJI	72.4	87.9

Table 1 shows no significant differences in Constant, UCLA and ASES between group A and group B. Significant difference is present in ACJI evaluation in favour of the group B, (*P* < 0.05).

The subjective evaluation revealed that 15% of the conservative treatment group rated their results as excellent, 85% as good and none as fair or poor. In the surgically treated group, 88% rated their results as excellent and 12% as good (Table 2).

**Table 2:** **Subjective evaluation of the injured shoulder**

	**Group A**	**Group B**
Poor	-	-
Fair	-	-
Good	85%	12%
Excellent	15%	88%

Data show better results in patients surgically treated with a significant higher satisfaction in group B (*P* < 0.05).

When patients were asked to compare the function of the injured shoulder to the contralateral healthy shoulder by assigning a percentage, 37% of group A patients judged that recovery was >90 compared to the healthy side and 63% assigned a recovery of 70%–80% compared to the contralateral shoulder. In group B, 67% of patients assigned a recovery >90% compared to the healthy side and 33% assigned a recovery of 70%–80% compared to the contralateral side (Table 3).

**Table 3:** **Function (%) of injured shoulder compared to the contralateral**

	**Group A**	**Group B**
70%–80%	63%	33%
>90%	37%	67%

Patients of group B show a better restore of shoulder function than patients of group A (*P* < 0.05).

Finally, the patient’s aesthetic subjective satisfaction was recorded. In group B, 78% of patients were highly satisfied, 22% were satisfied and none were poorly satisfied or unsatisfied, whereas in group A, no patients were highly satisfied, only 50% were satisfied, 30% were poorly satisfied and 20% were unsatisfied (Table 4).

**Table 4:** **Patient’s aesthetic subjective satisfaction**

	**Group A**	**Group B**
Unsatisfied	20%	-
Poorly satisfied	30%	-
Satisfied	50%	22%
Highly satisfied	-	78%

Patients of group B show a significant higher satisfaction at the final follow-up than patients of group A (*P* < 0.05).

### Radiological evaluation

The statistical analysis of our data demonstrated significant differences between the two groups in all measurements (Table 5).

**Table 5:** **Radiological results**

	**Group A**	**Group B**	***P*** **value**
ACD injured side T0	14.4 mm ± 5.4	14.2 mm ± 1.8	-
ACD healthy side T0	3.9 mm ± 0.9	3.4 mm ± 1.9	-
CCD injured side T0	20.3 mm ± 2.7	21.8 mm ± 3.5	-
CCD healthy side T0	9.9 mm ± 1.6	9.7 mm ± 2.4	-
ACD T1	10.2 mm ± 2.2	4.2 mm ± 1.2	-
CCD T1	16.1 mm ± 0.5	10.4 mm ± 0.2	-
Δ ACD	4.2 mm	10 mm	<0.05
Δ CCD	4.2 mm	11.4 mm	<0.05
*α* ACD	6.3 mm	0.8 mm	<0.05
*α* CCD	6.2 mm	0.7 mm	<0.05

This table shows the acromion clavicular distance (ACD) and coraco clavicular distance (CCD) of group A and group B. All the analysed parameters show significant difference in favour of the group B (*P* < 0.05).

*Δ* difference between the distance in mm at the final FU (T1) and T0 in the injured shoulder, *α* side to side difference in mm at final FU (T1).

### Complications

None of our patients developed a major complication, but in group B, these minor complications were detected: dislocation of the TightRope in one patient and calcifications in 70% of patients; all without any clinical correlation. A patient with a superficial wound infection that was treated with antibiotics for a month and healed and two patients with moderate osteolysis were recorded. In group A, calcifications were detected in 30% of patients and poor satisfaction with cosmetic results in 50% of patients.

### Return to sport activities

All patients returned to their sport/recreational athletic activities and their work. In group A, 80% of patients returned to the same level of physical activity as before the injury, whereas 20% were forced to reduce their activities. In group B, 83% of patients returned to the same level of physical ability as before the injury, whereas 17% were forced to reduce their activities. These frequencies did not differ significantly between the two groups (Table 6). Group A returned to sports after a mean of 60 days, whereas group B returned to sports after a mean of 120 days.

**Table 6:** **Study population**

	**Group A**	**Group B**	***P*** **value**
Number	25	30	n.s.
Male/female	25/0	30/0	n.s.
Mean age	28.5	29.2	n.s.
Sport activity level	Recreational	Recreational	n.s.
Return to sport	100% (80% same level)	100% (83% same level)	n.s.

## Discussion

The treatment of acute ACJ Rockwood type III dislocation is still debated because of disagreement regarding its optimal management. Moreover, current reviews have identified >150 different surgical techniques for its treatment [32,33]; in fact, there is still no gold standard for its treatment. Better subjective and radiological results were observed after surgical reduction of ACJ type III dislocation in young active patients. Furthermore, results similar to ours were recently reported by Korsten [23]. Comparison of our data with the international literature is challenging because of few other studies compared surgical and conservative treatment. Moreover, the majority of these reported different surgical techniques using different clinical scales. No differences were found in three objective clinical scales (Constant, ASES and UCLA) between the two groups; however, the ACJI score revealed better results in surgically treated patients. Notably, this scale assigns 35 of 100 points to the radiological evaluation and, in our opinion, uses criteria that are more sensible. Our objective results resemble those of other studies [10,31,34] even though the objective scales that were used by the other studies often differed from those of our study. The subjective evaluation revealed better results in group B for all subjective clinical scales, including higher satisfaction with cosmetic and functional outcomes in surgically treated patients. Now, the cosmetic outcome is becoming increasingly important. As presented in the above results, the cosmetic outcome, defined as permanent, prominent dislocation of the shoulder, was worse after conservative treatment.

In addition, the radiological evaluation showed better results for CCD and ACD measurements in surgically treated patients. Our results are similar to those of other authors, regardless of the surgical technique. In fact, at the final follow-up, in group B, a significant reductions in CCD and ACD were found, with values similar to the contralateral healthy shoulder and a low rate of complications.

Use of any surgical technique, if compared with conservative treatment, has a higher incidence of complications. In our study, none of our patients developed a major complication, but some minor complications were detected: mobilisation of the TightRope in one patient and coraco-clavicular ligament calcifications in 70% of patients without corresponding clinical findings. One case of infection and two cases with moderate osteolysis of the clavicle were recorded; none of them required revision surgery or hardware removal. In our opinion, surgical reduction during the acute phase with Tightrope could maintain the AC joint reduction during the first months and thereby enable biological healing, by serving work as an “internal brace” that keeps the joint reduced during the necessary healing time. Furthermore, this surgical technique allows visualisation of the achieved reduction, which could explain the low complication rate.

Recently, arthroscopic techniques have emerged and several relevant studies have reported good to excellent clinical and radiological results [35,36]. The major advantage of these arthroscopic procedures is that, if necessary, concomitant shoulder injuries can be detected and addressed at the same time, with minor cosmetic damage. However, complications caused by an incorrect placement of the tunnels in the coracoid process, leading to a dislocation of the Tightrope or a fracture of the coracoid process, have been described [37]. Therefore, this technique is restricted to experienced shoulder arthroscopists [35].

Recently, some authors described a two TightRope arthroscopic technique, using small variations of the TightRope fixing system and introducing anatomic reconstruction with two tunnels. Anatomical two-tunnel reconstruction with tendon grafts or synthetic materials seems appealing because it has been shown by biomechanical studies to restore the strength of the original two ligaments (the conoid and trapezoid), to produce an ultimate failure load that is equivalent to that of native CC ligaments [38,39] and to achieve significantly higher stability in the superoinferior and anteroposterior planes when compared to the native CC ligaments [36,40,41]. However, this technique is technically challenging and theoretically increases the risk of fracture [42]. Moreover, authors [35] confirmed that anatomic reconstruction could allow better clinical and radiological results. However, when they performed this technique, they observed a high dislocation rate in the first years. Because this arthroscopic procedure can lead to some difficulties, it is restricted to experienced arthroscopic shoulder surgeons. Our open, non-anatomic technique seems to be a simpler procedure that affords immediate visual control of the hole placement and joint reduction. Of note, several prior studies reported results of using the TightRope system in both acute and chronic patients for higher levels of dislocations than type III [43-45]. In contrast, a more uniform patient mix with respect to the type of dislocation and the timing of the intervention was included in our study. The results of the functional shoulder evaluation score did not differ significantly by treatment group except for the score that combines radiographic and subjective outcomes, the ACJI. Although it is not possible to state that surgically treated patients reported a better result, the high percentage of unsatisfied patients among those who were treated conservatively merits consideration. In fact, patients in the conservative group had high scores in the scales and early recovery of the sport activities, comparable to the surgical group; however, at the final follow-up, they reported a constantly present discomfort in the injured side. The scale for the sick scapula syndrome, presented by Kibler et al. [46] and Burkart et al. [47] and analysed by Gumina [48], could explain, as did the ACJI [25], this difference between the groups. Unfortunately, this scale was not analysed in our study.

Our study has some limitations. Most notable, it is a retrospective study with the biases inherent to this kind of design. Moreover, the surgical solution is more expensive than the conservative treatment and our study lacks cost analysis. Because the results of our study indicate distinct advantages of both treatment approaches, it is challenging to make definitive recommendations. Both the conservative and surgical treatments seem to offer excellent clinical results. The more rapid return to athletic activities following conservative treatment is a clear, short-term advantage of the conservative approach. However, the patient who chooses conservative treatment should be informed that in the future, he could feel a difference in the injured shoulder, which could lead to dissatisfaction with the conservative treatment.

## Conclusions

This retrospective comparison of conservative and surgical treatment for type III acromioclavicular joint dislocation shows better subjective and radiographic results for the surgically treated group. However, the objective outcome scores do not indicate significant differences between the two groups. Therefore, treatment options should be thoroughly discussed with patients, weighing all subjective, objective and radiographic outcomes and the relative advantages of each option.
